# Clinical Efficacy of Psychotherapeutic Interventions for Post-Traumatic Stress Disorder in Children and Adolescents: A Systematic Review and Analysis

**DOI:** 10.3390/children11050579

**Published:** 2024-05-11

**Authors:** Evgenia Gkintoni, Elias Kourkoutas, Vasiliki Yotsidi, Pilios Dimitris Stavrou, Dimitra Prinianaki

**Affiliations:** 1 Department of Psychiatry, University General Hospital of Patras, 26504 Patras, Greece; 2 Department of Primary Education, Research Center for the Humanities, Social and Education Sciences, University of Crete, 74100 Rethymno, Greece; eliaskourk@uoc.gr; 3 Department of Psychology, Panteion University, 17671 Athens, Greece; v.yiotsidi@panteion.gr; 4 Department of Psychology, University of Athens, 15784 Athens, Greece; pstavrou@psych.uoa.gr; 5 Department of Psychology, University of Crete, 74100 Rethymno, Greece; prinianakidimitra@gmail.com

**Keywords:** PTSD, trauma, psychotherapy, intervention, children, adolescents

## Abstract

*Background:* This systematic review aggregates research on psychotherapeutic interventions for Post-Traumatic Stress Disorder (PTSD) in children and adolescents. PTSD in this demographic presents differently from adults, necessitating tailored therapeutic approaches. In children and adolescents, PTSD arises from exposure to severe danger, interpersonal violence, or abuse, leading to significant behavioral and emotional disturbances that jeopardize long-term development. The review focuses on describing PTSD within two age groups, children (6 to 12 years) and adolescents (12 to 18 years), while evaluating the effectiveness of various clinical interventions aimed at this condition. *Methods:* Utilizing the PRISMA guidelines, this review systematically examines studies that assess clinical interventions for PTSD in the younger population. *Results:* Key symptoms of PTSD in children and adolescents include avoidance, overstimulation, flashbacks, depression, and anxiety. The review identifies several effective treatments, including Cognitive Behavioral Therapy (CBT), Trauma-Focused CBT (TF-CBT), Eye Movement Desensitization and Reprocessing (EMDR), Systemic Therapy, Play Therapy, Exposure Therapy, Relaxation Techniques, and Psychodynamic Psychotherapy. Particularly, TF-CBT is highlighted as the most effective and commonly used method in treating childhood and adolescent PTSD, as supported by most of the studies reviewed. *Conclusions:* A significant outcome of this study is the short-term effectiveness of CBT in reducing PTSD symptoms in children and adolescents. The findings underline the importance of psychotherapeutic interventions and mark a substantial advancement in understanding PTSD in young populations. It is crucial for practitioners to integrate various psychotherapeutic strategies into their practice to improve patient outcomes and treatment efficacy.

## 1. Introduction

*Trauma* is a unique situation that can result in personal anguish and hopelessness. Children may exhibit psychological responses to frightening situations that lead to immediate, acute, and sometimes chronic disruptions. Post-Traumatic Stress Disorder (PTSD) is a syndrome that occurs after experiencing a traumatic event that leads to intense fear and feelings of hopelessness, abandonment, or terror. It is classified under the category of Disorders Induced by Trauma and Stressors [1,2,3]. PTSD is caused by a traumatic event not typical of the human experience. Trauma is derived from the ancient Greek titrosko, which means to hurt. The medical definition of *trauma* is the “rapid or violent disruption of the continuity of the skin’s tissues, typically resulting in bleeding”. When the pressure exceeds the physiological tolerance limits of the body, physical damage occurs. When one person experiences an extremely stressful event on a mental level, inner psychic powers are mobilized to process it as efficiently as possible. If this procedure fails, internal cracks are produced. A psycho-traumatic event is any event that endangers a person’s life and causes severe psychological consequences and adjustment problems, even in those with no history of psychopathology.

When one person experiences an extremely stressful event on a mental level, inner psychic powers are mobilized to process it as efficiently as possible. If this procedure fails, internal cracks are produced. A psycho-traumatic event is any event that endangers a person’s life and causes severe psychological consequences and adjustment problems, even in those with no history of psychopathology.

Nevertheless, not all individuals who experience such a situation inevitably develop PTSD. PTSD is primarily a disorder characterized by the presence of multiple clusters of symptoms. Traumatic memories, hyperarousal, and a negative disposition characterize it. PTSD often occurs alongside melancholy or anxiety disorders [4]. Rapid transformations and extreme unpredictability characterize the modern era. Hence, it is common for children and adolescents to encounter highly stressful situations, and in some cases, even traumatic, thereby challenging their capacity to cope. These scenarios may encompass natural and human calamities, victimization, acts of violence, or unforeseen accidents. The aforementioned traumatic experiences have a significant impact on the physical, mental, and emotional well-being of children and adolescents, thereby exerting a profound influence on their overall development, formation of personality, and the subsequent trajectory of their future lives [5,6]. In addition to acute trauma, a series of other terms have been introduced in clinical theory and research in order to capture and describe the variety of child and adolescent traumatic experiences which may lead to PTSD. Depending on the nature, conditions, and effects of traumatic experiences, terms such as *developmental*, *relational/interpersonal trauma*, *complex*, *accumulated trauma*, as well as *covert* and *transgeneretional trauma*, refer to the complex nature of traumatic experiences in sensitive developmental periods that may produce a PTSD condition [7,8].

*PTSD* is a debilitating mental health condition that can affect traumatized children and adolescents. Among its symptoms are intrusive thoughts, nightmares, avoidance behaviors, and hyperarousal. The advancement of psychotherapeutic interventions plays a crucial role in addressing the symptoms of PTSD and facilitating the process of recovery among individuals affected by this condition. CBT is one of the most extensively researched and effective treatments for PTSD in children and adolescents. It involves identifying and challenging negative thoughts and beliefs associated with the traumatic event, as well as teaching coping skills to manage distressing symptoms. It has been demonstrated that CBT is effective in reducing PTSD symptoms in a variety of populations, including refugee children, those exposed to community violence, and those who have experienced single-incident traumatic events. Eye movement desensitization and reprocessing (EMDR) is another commonly used psychotherapeutic intervention for PTSD.

*EMDR* combines elements of exposure therapy and cognitive restructuring with bilateral stimulation, such as eye movements or tapping, to help individuals process traumatic memories and reduce distressing symptoms. EMDR has been found to be effective in reducing PTSD symptoms in children and adolescents, and it is advantageous for those who struggle to verbalize their traumatic experiences [9]. Other psychotherapeutic interventions have shown promise in the treatment of PTSD in children and adolescents, in addition to CBT and EMDR. Trauma-focused cognitive-behavioral therapy (TF-CBT) is a form of CBT that employs trauma-focused interventions, such as exposure therapy and narrative techniques, to address the effects of trauma on thoughts, emotions, and behaviors [10]. It has been determined that TF-CBT is effective in reducing PTSD symptoms and enhancing overall functioning in this population. Research has shown that it is crucial to take into account the unique experiences and requirements of children and adolescents when providing psychotherapeutic interventions for PTSD. Age, stage of development, and cultural background can impact the efficacy of treatment approaches. For instance, art-based interventions and group therapy have proven effective in reducing PTSD symptoms in refugee children and adolescents, particularly when language barriers are present. These interventions offer nonverbal means of expressing and processing traumatic experiences, which can be especially beneficial for individuals who struggle with verbal expression. Despite the availability of evidence-based psychotherapeutic interventions for the treatment of PTSD in children and adolescents, there are still limitations to their effective implementation [11].

In order to ensure the deliverance of effective interventions, it is also necessary to consider issues pertaining to therapeutic adherence and competence [12]. In addition, it is important to consider the effect of family factors, such as lack of parental support and family conflict, on the development and perpetuation of PTSD symptoms in children and adolescents [13]. Psychotherapeutic interventions are essential for the treatment of PTSD in infants and adolescents. CBT, EMDR, and TF-CBT are proven effective interventions for reducing PTSD symptoms and enhancing overall functioning in this population. Art-based interventions and other culturally sensitive approaches should be considered to address the unique requirements of individuals from diverse backgrounds. However, additional research is required to investigate the influence of therapist traits, family factors, and treatment adherence on treatment outcomes. Each intervention model brings unique benefits, targeting specific aspects of PTSD symptomatology. Moreover, the effectiveness of these interventions can be influenced by several factors, including the developmental stage of the child or adolescent and cultural considerations. Understanding these influences is crucial for tailoring interventions that are not only effective, but also culturally sensitive and developmentally appropriate.

The aim of this study is to comprehensively evaluate the efficacy of various intervention models for PTSD in children and adolescents, assessing their impact in ameliorating symptoms and improving life quality and to delineate their effectiveness and highlight factors contributing to successful outcomes, thereby informing practitioners about optimal strategies for treating young individuals afflicted by trauma.

## 2. Literature Review

According to an increasingly number of findings [14,15] and research on kidnapped and held hostage children indicated that these age groups could also develop PTSD following a traumatic event. Although the road to officially recognizing the disorder as a disorder of childhood and adolescence was long, it became apparent during the 1987 revision of the DSM’s third edition that all age groups are susceptible to it. This observation prompted a shift in attention towards the presentation of PTSD symptoms in children and adolescents, as it is apparent that they employ psychological and cognitive processes that differ from those observed in adults. The conventional perspective that a child’s reaction to traumatic events is a transient adjustment has been supplanted by the concept that trauma has enduring and profound developmental ramifications [16]. Currently, it is understood that PTSD presents itself in various ways among different age cohorts and necessitates distinct therapeutic approaches.

### 2.1. Clinical Signs and Symptoms of Trauma in Children and Adolescents

Psycho-traumatic events produce a variety of responses in children and adolescents. A Traumatic experience can disorganize and disrupt children’s psychosocial functioning and internal sense of cohesion, hindering their development if the traumatic experience is too harmful or repetitive. 

Delayed onset PTSD can occur months or years after a traumatic event [17,18]. Increased excitability and avoidance of trauma are the main reactions. Many people have unwanted thoughts about the event. Random sounds can cause reliving. Images can enter a child’s consciousness during quiet times, like before bedtime, disrupting sleep. Excitability often causes irritability, concentration issues, insomnia, disturbed sleep, and hypervigilance. Elevated arousal, memory, and concentration issues can hurt school performance [6]. 

Age and, to a lesser extent, gender predict trauma reactions. According to [16], random trauma causes despair. Generalized anxiety and obsessions are common. Younger children are more aggressive and destructive, and may play or draw with traumatic event content and behaviors. Young children with chronic stress may develop behavioral or attachment disorders. Younger children often regress to earlier developmental stages like bedwetting or verbal loss [19,20]. After 8–10 years, adolescents’ responses resemble adults’. School-aged children can understand and make sense of a situation, including the long-term effects of trauma and their role in it. Adolescence emphasizes trauma’s long-term effects. Its social effects are also emphasized [14]. 

Adults react to trauma with dread, horror, or despair, whereas children may display disorganized and disturbed behavior. Avoidance behaviors are also more difficult to observe in children because they are frequently not cognitively aware of their presence. Loss of interest is another difficult-to-observe behavioral parameter in children, as it typically manifests as listless play, daydreaming, or increased use of imaginary play. Physical distress is common among children and adolescents who have experienced a traumatic event [16]. In addition to age-based differences, gender-based differences emerge as a growing number of females are diagnosed with PTSD [14]. 

Regarding children (6–12 years old), previous research shows that school-aged children rarely have visual “flashbacks” or amnesia after traumatic events. They seem to have a disturbed sense of time (time asymmetry), where they cannot recall the correct temporal order of trauma events, or “omen generation”, the belief that omens foretold the trauma. They are constantly vigilant due to this perception. The above symptoms are rare in adults [16]. Children are less emotionally detached than adults [6,14]. This age group uses “post-traumatic play”, re-enacting psycho-traumatic events. This play is traumatic and increases the child’s anxiety and tension, not helping him reduce negative emotions. They depict the event in drawings, plays, and other verbal and nonverbal ways [21]. The symptoms may appear at school and at home. They may cause nightmares, insomnia, sleepwalking, or bedwetting at home. As the child expects these symptoms to occur outside of bedtime, they may be afraid to sleep alone or have other problems. School issues may include agitation, hyperactivity, inability to concentrate, and behavioral or academic issues. These symptoms resemble ADHD, which is often misdiagnosed [16]. 

For adolescents (12–18), although their clinical picture is more like adults’, there are still differences. Teens can develop dissociative symptoms, angry outbursts, self-harm, and substance abuse after repeated trauma. Teens are especially vulnerable to trauma. The importance of peer groups and this developmental period can increase risky behavior and the disorder’s long-term adverse effects [16,17,21]. Teenagers, like younger children, may act out trauma in daily life. After a traumatic event, this age group is more likely to “dramatize” the trauma and incorporate it into their daily lives [20]. They have “survivor’s guilt” when they feel partially responsible [16,19]. Trauma mainly affects adolescents academically and socially. Teens with PTSD are three times more likely to attempt suicide than those without PTSD [19].

### 2.2. Navigating the Complexity: Diagnosing PTSD in Children and Adolescents 

The diagnosis of PTSD in children and adolescents is complicated in ways that are absent in the diagnosis of PTSD in adults. Difficulties arise at various stages of the diagnostic procedure, such as when defining a psycho-traumatic event in children and adolescents or anticipating the onset of symptoms. On the one hand, children may exhibit cognitive deficits and limitations in expression or verbalization, which may hinder their comprehension of the psycho-traumatic event [16]. In the case of children, there are frequently difficulties in reporting certain reactions; for instance, they frequently have trouble reporting correct instances of avoidance reactions because they may be too difficult to verbalize or comprehend and require a more complex cognitive introspection [14]. It is also not uncommon for them to avoid discussing the event out of fear of upsetting their parents or out of concern that they will be rejected or not understood.

On the other hand, it is well known that infants have a vivid imagination, which they use to embellish their accounts of their experiences. There is also a tendency to mistake the actual for the ‘appearance’. A situation may not be particularly violent, threatening, or traumatizing to adults, but it can still cause significant trauma to children, such as when they are disoriented in a crowd of strangers or when they are sexually touched [16,19,20,21].

Given the complexity and individual variability in PTSD symptoms among youth, this review critically examines a spectrum of approaches, including Cognitive-Behavior Therapy (CBT), Trauma-Focused CBT, Prolonged Exposure Therapy, Eye Movement Desensitization and Reprocessing (EMDR), Narrative Exposure Therapy, Play Therapy, Systemic Trauma Therapy, and Psychodynamic Therapy. These interventions are evaluated based on their theoretical foundations, empirical support, and adaptability to different ages, traumas, and cultural backgrounds. Below is a very brief description of the conventional psychotherapeutic approaches used to be applied for the treatment of PTSD.

### 2.3. Cognitive-Behavior Therapy (CBT) 

CBT is the most studied psychotherapeutic intervention for PTSD in children and adolescents, showcasing effectiveness across various trauma types. It combines behavioral and cognitive techniques to modify dysfunctional thoughts and behaviors related to trauma. Key strategies include psycho-education, relaxation, exposure to trauma in controlled settings, and cognitive restructuring. Studies highlight the importance of factors such as age, ethnicity, and parental involvement in treatment efficacy. School-based programs like Cognitive-Behavioral Intervention for Trauma in Schools (CBITS) have also been effective, particularly in fostering social-emotional well-being in affected children.

### 2.4. Trauma-Focused CBT (TF-CBT) 

TF-CBT, a variant of CBT specifically tailored for trauma, has shown superior efficacy, particularly for victims of sexual abuse, but is also adaptable for other traumatic experiences. This structured, brief model emphasizes skill development, trauma exposure, and cognitive processing, often involving parents to enhance treatment stability and effectiveness. The therapy spans approximately 12 to 18 sessions and utilizes a multi-component approach including psycho-education, parenting skills, emotional regulation, and cognitive coping strategies.

### 2.5. Prolonged Exposure Therapy 

Since its development in 1982, prolonged exposure therapy, another form of CBT, has been a prominent method for treating adolescent PTSD. It involves safe exposure to traumatic content to reduce avoidance behaviors and emotional numbing associated with PTSD. The approach is based on emotional processing theory, aiming to alter pathological associations with trauma through repeated exposure sessions.

### 2.6. EMDR Therapy

Eye Movement Desensitization and Reprocessing (EMDR) therapy, developed by Francine Shapiro, is used for children and adolescents with PTSD, utilizing rhythmic eye movements to process and desensitize trauma. Despite limited randomized controlled trials in children, EMDR has been effective, especially in settings that ensure a stable therapeutic relationship and proper relaxation techniques.

### 2.7. Narrative Exposure Therapy 

This therapy focuses on constructing a chronological narrative of the individual’s life to contextualize and process traumatic events. It is particularly useful for individuals with complex trauma histories, such as refugees, and typically lasts between 5 to 10 sessions. The approach integrates psychoeducation and direct trauma processing.

### 2.8. Play Therapy 

Play therapy provides a non-verbal medium for younger children to express complex emotions and thoughts. It is adaptable to the child’s cultural and developmental needs, often involving creative play, which helps children articulate and work through their difficulties. Research supports its efficacy, particularly when integrated with caregiver involvement.

### 2.9. Systemic Trauma Therapy 

Focusing on the child’s social environment and emotional regulation, this therapy addresses the broader systemic issues influencing a child’s trauma response. It includes legal advocacy, home care, and psycho-education, emphasizing a comprehensive approach to creating a stable, supportive environment for the child.

### 2.10. Psychodynamic Therapy 

While traditionally less emphasized in empirical research compared to other therapies, psychodynamic therapy focuses on resolving internal conflicts through understanding past experiences and their impact on present behavior. This method involves exploring unconscious conflicts, defense mechanisms, and the therapeutic relationship, and is shown to be effective especially for internalizing and affective disorders.

The literature underscores a diverse array of effective psychotherapeutic interventions for treating PTSD in children and adolescents, with cognitive-behavioral strategies being the most prevalent. Factors such as therapy duration, parental involvement, and adaptation to the child’s developmental stage play critical roles in the success of these interventions. Specifically, the current research will focus on analyzing the effectiveness of interventions across different developmental stages and cultural contexts, aiming to identify tailored strategies that optimize treatment outcomes. The research questions based on the systematic analysis revolve around understanding the clinical efficacy of psychotherapeutic interventions for Post-Traumatic Stress Disorder in children and adolescents are summarized below:

- [RQ1] How do various psychotherapeutic interventions, including TF-CBT, compare in effectiveness for treating PTSD in different pediatric age groups (children aged 6–12 and adolescents aged 12–18)?
*This question seeks to explore the comparative effectiveness of various psychotherapeutic interventions, including TF-CBT, for treating PTSD in distinct pediatric age groups: children aged 6–12 years and adolescents aged 12–18 years.*
- [RQ2] What role does parental involvement play in the effectiveness of psychotherapeutic interventions for PTSD in children and adolescents?
*This question investigates the impact of family engagement in the therapeutic process, which could provide insights into how treatment protocols might be enhanced by integrating family-based support.*
- [RQ3] How do cultural and developmental factors influence the efficacy of psychotherapeutic treatments for PTSD in children and adolescents?
*This question addresses the need to tailor psychotherapeutic interventions to the cultural backgrounds and developmental stages of young patients to optimize treatment outcomes.*
- [RQ4] What are the long-term effects of psychotherapeutic interventions on PTSD symptoms in children and adolescents, and how sustainable are these effects over time?
*This question focuses on the durability of treatment effects, crucial for understanding and improving the long-term care and support strategies for young individuals affected by PTSD.*
- [RQ5] What are the comparative effects of different psychotherapeutic interventions (CBT, TF-CBT, EMDR, etc.) on PTSD symptoms reduction in children versus adolescents?
*This question aims to explore how different psychotherapeutic interventions, such as CBT, TF-CBT, and EMDR, differentially impact the reduction of PTSD symptoms in children compared to adolescents.*
- [RQ6] How do developmental stages affect the choice and success of psychotherapeutic interventions for PTSD in children and adolescents?
  *This question seeks to understand how treatments should be adapted to match the developmental needs of children at different ages for optimal effectiveness*.

## 3. Materials and Methods

This paper focuses on English language articles, studies, meta-analyses, and reviews published between 2016 and 2023. The exclusion of pre-2016 studies is justified for several reasons. Firstly, psychotherapeutic interventions and methodologies evolve rapidly; thus, studies conducted after 2016 are more likely to incorporate the latest advancements in psychotherapy. These include new techniques or modifications to existing practices specifically tailored to better serve children and adolescents, ensuring that the review captures the most current evidence reflecting state-of-the-art practices.

Additionally, diagnostic criteria for PTSD and related conditions have likely been updated, particularly with the revisions in the DSM-5. Also, the nature and impact of traumatic experiences have evolved over the past five years due to various social, environmental, and global factors. Recent studies more accurately reflect the current trauma types experienced by children and adolescents, making the findings more applicable to contemporary clinical practice. Such research also addresses the specific developmental needs and symptom presentation variations across different ages within the children and adolescent populations, offering a nuanced understanding of how treatments can be tailored for different age groups.

Furthermore, the study excluded reports that were not retrievable due to language and publication restrictions. Specifically, studies not published in English or not accessible through the primary databases searched—Scopus, PsycINFO, PubMed, Web of Science—were omitted. This ensured that the review was limited to English language publications accessible through these databases, prioritizing quality and reliability.

More emphasis is placed on quantitative research that seeks to demonstrate the efficacy of therapeutic interventions used to treat childhood and adolescence PTSD and post-analyses to determine which are deemed more effective. The search was conducted to identify the most recent studies with adequate sample size and methodology. Preference was given to empirical and randomized controlled trials.

**Figure 1:**
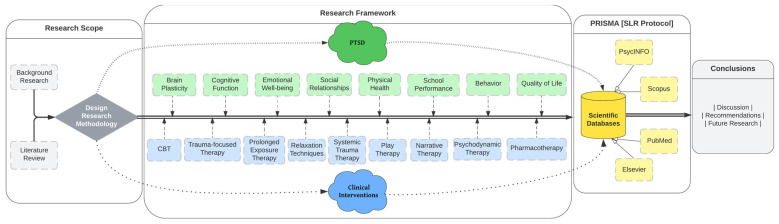
Flowchart of proposed research design.

However, their number is negligible as child–adolescent psychopathology is still in its infancy. Studies conducted with a sample of adults or small sample size (e.g., case studies) conducted before 2016 were disregarded. The PsycINFO, Scopus, PubMed, and Elsevier databases were searched using Boolean logic, following the PRISMA guidelines which outline the preferred reporting items for systematic reviews [22]: Children and adolescents with Post-Traumatic Stress Disorder and Post-Traumatic Stress Disorder Clinical Interventions for PTSD in Children and Adolescents, as well as PTSD in children and adolescents, PTSD treatment, and PTSD interventions in English. In addition to the general search with the terms listed above, a separate search was conducted for each intervention, such as cognitive-behavioral therapy, systems therapy, trauma system therapy, etc. (Figure 1). 

**Figure 2:**
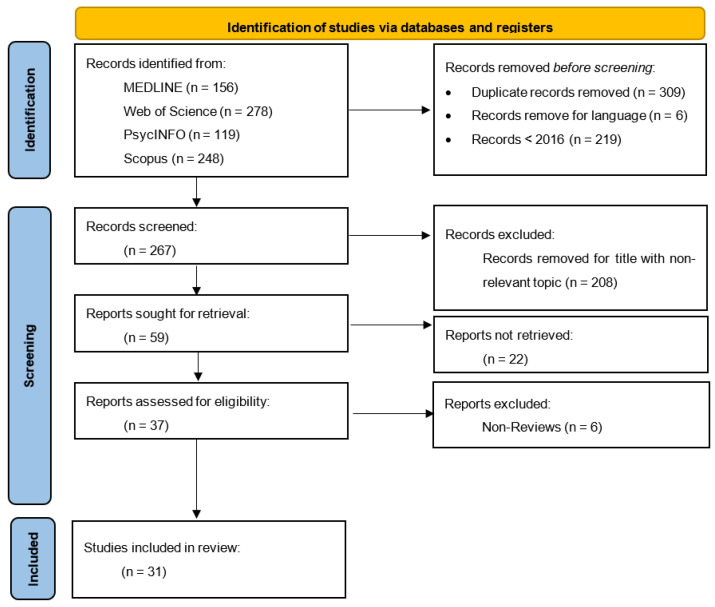
Flowchart of PRISMA Methodology.

At first, the investigation was carried out using broader criteria. Subsequently, excluding studies that failed to meet the predetermined, rigorous, and precise criteria commenced. The calculation of the survey date was a crucial factor. The holding date was strictly limited to the period after 2016. The sample’s age range was also essential, as it had to fall between 5 and 17 years old. The permissible deviation from the specified age was either two years or less. Nevertheless, most studies reviewed have a sample age range from 5 to 17 years. The databases incorporated the primary keywords “PTSD” and “Clinical Interventions”, as depicted in the flowchart provided (Figure 2). Quantitative research was prioritized over qualitative research. The rationale behind the particular study was to initially collect data on each approach, followed by a demonstration of their effectiveness. The search was conducted in major databases including Scopus, PsycINFO, PubMed, and Web of Science, using a set of specific keywords and filters to refine the search results. The keywords used in the search were “PTSD” and “Clinical Interventions”. These keywords were chosen to capture the broad scope of clinical interventions and psychotherapies applied within the field of PTSD in children and adolescents.

The use of Boolean operators (AND, OR, NOT) allows for the combination of these keywords to refine search results effectively. The strategy used was the following based on the query string:

*TITLE-ABS-KEY (ptsd AND clinical AND interventions) AND PUBYEAR > 2015 AND PUBYEAR < 2024 AND (LIMIT-TO (PUBSTAGE, “final”)) AND (LIMIT-TO (EXACTKEYWORD, “adolescent”) OR LIMIT-TO (EXACTKEYWORD, “child”) OR EXCLUDE (EXACTKEYWORD, “adult”) OR EXCLUDE (EXACTKEYWORD, “depression”) OR EXCLUDE (EXACTKEYWORD, “procedures”) OR EXCLUDE (EXACTKEYWORD, “international classification of diseases)) AND (LIMIT-TO (LANGUAGE, “english”)) AND (LIMIT-TO (DOCTYPE, “ar”)) AND (LIMIT-TO (SUBJAREA, “psyc”)).*

Out of the initial pool of 801 bibliometric sources, a subset of 31 sources was selected for inclusion in this study. All subsequent evaluations encompassed qualitative and quantitative assessments of the effectiveness of the interventions. The clinical sample underwent evaluation utilizing the diagnostic criteria outlined in the DSM-5 or ICD-11 for the identification of Post-Traumatic Stress Disorder. An interview or questionnaire was typically conducted with either the infant or their caregiver. While a PTSD diagnosis was not mandatory for inclusion in the sample, the presence of PTSD symptoms was a requirement in most studies. The conventional experimental design consisted of one or more experimental groups, which varied depending on the number of interventions in each study, along with a control group that did not undergo the intervention. Typically, there was only a preliminary sample preparation after commencing the research. However, in numerous cases, subsequent studies were carried out to ascertain the long-term stability of the interventions’ effects. There is a scarcity of meta-analytic studies, systematic reviews, and randomized controlled trials that have been carried out to compare the effectiveness of interventions for teenage PTSD. Consequently, most reviews incorporate similar studies, necessitating their consideration when interpreting our findings.

## 4. Results

Most of the literature on PTSD treatment focuses on CBT, particularly trauma-focused CBT. 

### 4.1. Cognitive Behavioral Therapy (CBT) and Variants (TF-CBT, Group CBT)

Multiple studies indicate that trauma-focused cognitive-behavioral therapy (CBT) is the most efficacious intervention for PTSD in infants and adolescents. Based on a meta-analysis [21], trauma-focused cognitive-behavioral therapy (TF-CBT) is the most efficacious and widely recognized treatment intervention for PTSD in adolescents. According to the meta-analysis conducted by [23], TF-CBT emerged as the most efficacious intervention, exhibiting a substantial effect size compared to the absence of any treatment. This research aligns with the findings of previous studies, which also determined that TF-CBT is the most efficacious intervention for childhood and adolescent PTSD. The findings of a meta-analysis [23] indicate that EMDR therapy exhibited statistically significant effects in the uncontrolled analysis. In contrast, the controlled studies included in the analysis reported only minor to moderate impacts. Based on a study [24], CBT is the well-established treatment for PTSD in infants and adolescents. In contrast, all other treatments are considered potentially sufficient or under investigation. TF-CBT is widely recognized as the most productive and firmly established approach. Group CBT, commonly conducted in a school environment, was considered sufficient. However, group CBT involving parental involvement and EMDR therapy may yield positive results. 

A study [25] identifies several shared characteristics among the practical approaches that were identified: psycho-education on the frequency, effects, and management of trauma; instruction in regulating emotions and employing problem-solving techniques; exposure to simulated or real-life situations; and cognitive processing. Given the scarcity of research on psychopharmacological interventions, mental health professionals should exercise caution when employing medication. According to the study conducted by [26], there is a negative relationship between the amount of medical information that children are exposed to and their level of posttraumatic stress experienced several months after a medical incident. There is a notable correlation between preschoolers and children of school age. Furthermore, Ref. [21] has observed that six recent meta-analytic studies and systematic reviews have assessed psychological interventions for post-traumatic stress disorder (PTSD) in children and adolescents. The study supported CBT, EMDR, narrative exposure therapy, and interventions implemented in a classroom setting. CBT and TF-CBT have been recognized as established therapeutic approaches for PTSD in the pediatric and adolescent population. Despite having a more limited evidence base, there was also support for EMDR, narrative exposure therapy, and school-based interventions. 

The study conducted by [27] presents several significant findings. First and foremost, it showcases exercise training’s capacity to impact cortisol levels. Furthermore, it presents a cost-efficient group intervention for individuals suffering from PTSD. Exercise training is hypothesized to yield a distinct outcome compared to a placebo intervention.

The research findings [28] indicated that TF-CBT interventions exhibited greater efficacy compared to control conditions in terms of alleviating symptoms associated with PTSD. Adolescents who have undergone traumatic experiences may manifest psychiatric disorders, including affective, personality, and psychotic disorders, in addition to PTSD. These disorders can arise due to the enduring and immediate consequences of intricate traumatic incidents. These aforementioned factors have the potential to contribute to the emergence of dissociative and somatic symptoms, which may exhibit more significant debilitation compared to symptoms encountered after a traumatic incident. Personalized and appropriate therapeutic interventions are required to address the demand for trauma clinical interventions.

The research conducted by the authors [29] revealed that the implementation of a focused preventive intervention resulted in a significant reduction in the intensity of symptoms associated with PTSD as time progressed. After three months, it was observed that the intervention resulted in a more rapid decrease in post-traumatic stress symptoms (PTSS) severity scores among children who received the intervention as compared to those in the control group. The intervention demonstrated significant therapeutic outcomes in the diagnosis of PTSD, evaluation of functional impairment, and management of behavioral difficulties in young children who have experienced injury. The research provides promising preliminary findings regarding the efficacy of the targeted preventive intervention in facilitating recovery from PTSS in young children who have sustained injuries. This highlights the notable clinical ramifications associated with the provision of psychological assistance to young children and their parents following a traumatic incident.

Moreover, an independent study [30] presents the main findings of the research, which involve an analysis of variables that can forecast, assess, and manage PTSD in adolescents. The investigation additionally examines the potential for reviewing distinct attributes or domains, such as cognitive capacities, memory, and executive functioning, to enhance comprehension of PTSD and its ramifications. In addition, the paper suggests implementing a multidimensional methodology for examining PTSD and trauma.

TF-CBT can offer significant benefits in tackling the psychological and social difficulties experienced by young individuals who are susceptible to or engaged in familial sex trafficking and labor exploitation. TF-CBT has been found to effectively address the psychosocial challenges encountered by children who have undergone childhood adversity and trauma. Additionally, it has been suggested that TF-CBT may substantially impact their resilience.

Cognitive therapy is an effective treatment for children and young individuals with PTSD. Next, narrative exposure, play therapy, and other forms of individual TF-CBT are implemented [31]. Furthermore, it has been acknowledged that both individual modalities of TF-CBT and play therapy are cost-effective approaches for addressing PTSD in children and young individuals who experience PTSD symptoms more than three months following the traumatic incident. Furthermore, the cost-effectiveness of family therapy and supportive counseling compared to alternative interventions is highly unlikely.

According to the research conducted by [32], it was observed that college students who had previously encountered elevated levels of ADHD symptoms during their childhood exhibited a notably greater prevalence of trauma exposure and symptoms associated with PTSD. The implications of these findings extend to clinical interventions targeting children and adolescents, high school counseling, and accessibility services related to psychological well-being and academic adaptation. According to another study [33], interventions aimed at preventing trauma, PTSD, and depression should be thorough and targeted at different levels. These levels include the individual/interpersonal level, which involves reducing abuse within households and immediate surroundings, and the community/societal level, which consists of reducing crime rates in communities and improving conviction policies.

The initial findings of the first benchmarking study on TF-CBT are presented in the study [34]. This study investigates the effectiveness and applicability of TF-CBT in urban community settings that specifically target economically disadvantaged adolescents. The research conducted by [35] unveiled significant disparities between the initial and subsequent evaluations of symptoms related to depression and PTSD. Even though 62% of the participants experienced negative life events while participating in the program and were also going through the asylum process, this observation was made. A total of six categories were identified through the qualitative interviews, namely social support, normalization, valued tools, comprehensibility, manageability, and significance. The results are consistent with the program theory of Trauma-Related Therapy (TRT), which suggests that through the exchange of experiences in a safe and supportive environment, as well as the acquisition of coping strategies such as trauma-specific exposure and behavioral activation, the youth’s sense of coherence will be strengthened, leading to a reduction in the intensity of depressive and PTSD symptoms. The results of the study indicate that TRT exhibits potential as a preventative measure for individuals belonging to underrepresented minority groups (URMs) who manifest symptoms associated with PTSD.

The research by [36] provides preliminary evidence regarding the cost-effectiveness of cognitive therapy within this population. The intervention was conducted by clinical researchers, and replicating the findings in a broader clinical setting may present difficulties. From the perspective of the National Health Service (NHS) and personal social services, CT-PTSD emerged as a financially efficient alternative to conventional care. 

The publication referenced as [37] highlights the bidirectional impact of post-traumatic stress symptoms (PTSS) on both adolescents and their parents after a catastrophic event. The findings indicate that the PTSS of both mothers and fathers at 12 months can serve as a predictor for the occurrence of PTSS in adolescents at 18 months. The presence of PTSS in adolescents at 12 months was observed to be associated with maternal PTSS at 18 months, while no significant association was found with paternal PTSS.

The predominant focus of the randomized controlled trials (RCTs) was directed towards students and retirees residing in low- and middle-income countries (LMICs). These trials consistently exhibited effectiveness in alleviating symptoms of anger, improving life skills and overall functioning, as well as reducing the prevalence of post-traumatic stress disorder (PTSD), depression, and anxiety. In low- and middle-income countries (LMICs), there is significant potential for addressing mental health issues and other health-related areas by implementing comprehensive programs focusing on enhancing parent–child interactions and developing various life skills related to the individual and their social environment [38].

TF-CBT and CBITS have demonstrated significant clinical enhancement and enduring benefits. While there are empirically supported effective treatments for PTSD, most of them involve cognitive-behavioral approaches [39].

Moreover, psychosocial interventions have been shown to have a significant and beneficial effect on the outcomes of PTSD, depression, and anxiety in refugees and asylum seekers who are undergoing distress. Most evidence confirms the effectiveness of interventions based on cognitive behavioral therapies and incorporating a trauma-focused component. Previous research has been suggested to develop evidence-based guidelines and implementation packages [40].

Moreover, according to a study [41], TF-CBT is effective in reducing symptoms of post-traumatic stress and improving psychosocial functioning over time.

### 4.2. Eye Movement Desensitization and Reprocessing (EMDR)

EMDR has demonstrated efficacy in mitigating symptoms of PTSD, depression, and anxiety in comparison to alternative therapies and control treatments. Based on both controlled and uncontrolled studies, the suitability and effectiveness of this intervention are evident in its application to children and adolescents. The study conducted by researchers [23] revealed a significant association between EMDR and a reduction in symptoms of PTSD, depression, and anxiety, in comparison to alternative therapies and control treatments. Moreover, the systematic review reveals a growing body of empirical evidence that substantiates the clinical efficacy of EMDR as a therapeutic approach for addressing intricate childhood trauma across various age groups. The analysis encompassed six studies that demonstrated favorable outcomes for EMDR compared to non-specific therapy, CBT, fluoxetine, and control conditions. Nevertheless, the reliability of these findings exhibited variability, with specific disparities failing to attain statistical significance.

In addition, the research conducted by [42] introduces alternative therapeutic approaches for the treatment of trauma. The treatment approach consisted of mindfulness-based techniques, expressive arts, and group therapy utilizing EMDR. The results support the use of this intervention as a possible short-term integrative/complementary measure to reduce psychological distress in adolescents who have had multiple adverse childhood experiences (ACEs). Although there was an observed improvement in psychological well-being within the two months following release, the adolescents may require further group or individual assistance to reinforce and consolidate the mental health benefits obtained from this intervention.

According to a study conducted by researchers [43], Eye Movement Integration (EMI) has been identified as a potentially advantageous temporary therapeutic intervention for young children residing in resource-constrained environments. The study revealed a noteworthy reduction in all symptoms associated with post-traumatic stress except for one specific symptom.

### 4.3. Narrative Exposure Therapy (NET)

The efficacy of NET has been verified in clinical environments for children and adolescents who have undergone multiple traumatic experiences. The primary objective of this approach is to establish a cohesive life narrative encompassing various traumatic experience. The study’s main findings [44] demonstrate that both narrative exposure therapy (NET) and treatment as usual (TAU) led to a decrease in PTSD and psychological distress. Furthermore, an increase in resilience was observed within both groups. The reduction in symptoms of PTSD was particularly noteworthy exclusively in the NET group, demonstrating significant effect sizes. Similarly, there was a substantial decrease in the proportion of participants with PTSD at clinical levels, observed explicitly in the NET group. The study provides preliminary confirmation of the safety, effectiveness, and usefulness of NET in clinical environments for children and adolescents who have undergone multiple traumatic experiences.

Additionally, the study’s main findings [44] indicate that TF-CBT yielded a significant reduction in symptoms of PTSD, with only 1 out of 16 participants meeting the diagnostic criteria after the treatment. Furthermore, the utilization of self-report measures revealed noteworthy improvements in the levels of PTSD, anxiety, and depression. While a minority of participants encountered a transient worsening of symptoms during certain phases of the treatment, all symptoms were wholly alleviated by the conclusion of the treatment. Furthermore, a significant proportion of the participants reported that the intervention yielded positive outcomes.

### 4.4. Specialized Interventions (PE-A, RRFT, and Systemic Therapy)

The study conducted by researchers [45] revealed that both Prolonged Exposure Treatment for Adolescents (PE-A) and supportive counseling (SC) yielded significant improvements in symptoms of PTSD and depression throughout treatment. However, it was observed that the participants in the PE-A group maintained their progress in PTSD and depression assessments throughout the 12-month follow-up period, indicating the long-lasting effectiveness of PE-A. The study additionally demonstrated that the PE-A protocol is applicable in a South African setting when implemented by novice counselors within a school setting.

A study [46] found that prolonged exposure (PE-A) was more effective than supportive counseling in mitigating symptoms of PTSD in adolescents. A notable difference in progress was observed between the two groups during the post-intervention, 3-month, and 6-month follow-up evaluations. In the aforementioned study, a significantly more significant proportion of participants in the PE-A group exhibited a ‘good response’ compared to those in the supportive counseling group, indicating a greater likelihood of favorable treatment outcomes with PE-A. In the treatment of PTSD in adolescents, both Prolonged Exposure Therapy for Adolescents (PE-A) and supportive counseling are effective. Nevertheless, PE-A resulted in a considerably more significant decrease in PTSD symptoms and a greater likelihood of attaining remission in comparison to supportive counseling.

The study’s findings [47] showed that RRFT (risk reduction through family therapy) yielded notably greater reductions in the duration of substance use at both the 12-month and 18-month intervals compared to the control group.

Additionally, it is worth noting that both the RRFT and TAU groups demonstrated significant reductions in symptoms of post-traumatic stress disorder (PTSD) at months 3, 6, 12, and 18 in comparison to their initial condition. Furthermore, no noticeable differences were observed between the two groups. In neither condition was there any observed evidence of a worsening of substance use issues.

Ultimately, the study [48] revealed that every child or adolescent has experienced at least one traumatic event related to war, which is associated with increased mental health and behavioral issues. The prevailing conditions require counseling programs tailored to assist these families and children. The study’s findings suggest incorporating parental emotional validation and invalidation as treatment objectives in clinical intervention practices for this population could yield positive outcomes. Interventions propose that therapists utilize emotional validation techniques to foster a more potent therapeutic alliance.

## 5. Discussion

The systematic review on PTSD in children and adolescents underscores the effectiveness of several psychotherapeutic interventions, with a particular emphasis on the efficacy of TF-CBT and EMDR. These interventions are highlighted for their robust outcomes in reducing PTSD symptoms, improving emotional and behavioral functioning, and addressing comorbid conditions such as anxiety and depression. The review also points to the potential benefits of other therapeutic approaches, including play therapy, exposure therapy, and relaxation techniques, underscoring the vast array of options available for treating PTSD in young populations. The findings emphasize the necessity for interventions to be adaptable to the individual’s specific needs, considering factors such as age, developmental stage, trauma type, and cultural background to optimize treatment efficacy.

Furthermore, the present study indicates the importance of incorporating the child’s social environment into the therapeutic process, noting the positive impact of family involvement and school-based interventions on treatment outcomes. This holistic approach supports not only the individual’s recovery from PTSD, but also their overall development and well-being. However, the review acknowledges a notable gap in research on pharmacological treatments for PTSD in this demographic, suggesting a cautious approach to medication and a preference for psychotherapeutic interventions. The call for further research highlights the need for long-term studies on the outcomes of these interventions, their effectiveness across different cultural contexts, and the management of comorbid conditions. Overall, the systematic review offers valuable insights into the current state of treatment for PTSD in children and adolescents, advocating for evidence-based, tailored, and accessible psychotherapeutic interventions to meet the diverse needs of this vulnerable population.

### 5.1. Research Question 1 [RQ1]

The studies relevant to [RQ1] provide valuable insights into the effectiveness of psychotherapeutic interventions for treating PTSD in children aged 6 to 12 years and adolescents aged 12 to 18 years. In study [33], researchers conducted an analysis of various psychotherapy forms, highlighting cognitive therapy as the most cost-effective option across specified age groups. In the study [29] the research team conducted a systematic review and meta-analysis specifically assessing TF-CBT, revealing its superiority in reducing PTSD symptoms compared to control groups in children and adolescents. Furthermore, the study [42] focused on Narrative Exposure Therapy (NET), demonstrating significant reductions in PTSD symptoms and psychological distress, enhancing resilience in multiply traumatized children and adolescents. Also, researchers in the study [26] explored the impact of medical information exposure on post-traumatic stress in children, offering insights into how different age groups respond to intervention methods. In the study [40], the researchers analyzed the clinical efficacy of various interventions across a broad age range, including children and adolescents, providing a comprehensive view of their outcomes in treating PTSD.

Also, ref. [RQ1] delves into comparing TF-CBT with other psychotherapeutic approaches in reducing PTSD symptoms among children and adolescents who have experienced traumatic events. The researchers in the study [29] conducted a systematic review and meta-analysis focusing on TF-CBT, affirming its superiority in reducing PTSD symptoms compared to control groups, suggesting a notable advantage over alternative therapeutic methods. Researchers in the study [40] analyzed various interventions, including TF-CBT, and highlighted its effectiveness compared to other therapies like EMDR and play therapy across different age groups. Researchers in the research project [48] shed light on TF-CBT’s efficacy alongside EMDR and narrative exposure therapy, particularly in reducing PTSD symptoms among war-affected youth. Researchers in the study [42] contextualized TF-CBT’s effectiveness within a broader therapeutic landscape by comparing its outcomes with Narrative Exposure Therapy (NET) in clinical settings. Researchers in the study [39] provided a comparative analysis, reinforcing TF-CBT’s significant improvement and sustainability in benefits compared to other therapies. Researchers in the study [49] indirectly contributed to the comparative landscape by evaluating Prolonged Exposure Therapy for Adolescents (PE-A) against supportive counseling. Researchers in the study [47] added insights into TF-CBT’s comparative benefits within a broader treatment context, despite their primary focus on risk reduction through family therapy. Furthermore, researchers in the study [33] provided a cost-effectiveness analysis, highlighting TF-CBT’s economic and therapeutic advantages over other interventions. These studies collectively underscore the effectiveness of TF-CBT in reducing PTSD symptoms in children and adolescents compared to alternative approaches.

### 5.2. Research Question 2 [RQ2]

[RQ2] focuses on the role of parental involvement in psychotherapeutic interventions for children and adolescents with PTSD. Danielson et al. (2020) demonstrated that family therapy, which includes parental engagement, resulted in greater reductions in substance use and significant decreases in PTSD symptoms over time. Their findings emphasized the importance of involving parents in the therapy process to improve treatment outcomes in adolescent populations. Additionally, the studies [37,50,51] also contribute to this aspect of PTSD treatment. These studies collectively showcase the positive impacts of integrating parental involvement into therapy for children and adolescents with PTSD, highlighting its potential to enhance the efficacy of psychotherapeutic interventions.

### 5.3. Research Question 3 [RQ3]

[RQ3] delves into the influence of cultural and developmental factors on psychotherapeutic interventions for treating PTSD in children and adolescents. Luoni et al. (2018) emphasize the importance of individualizing trauma treatment by considering cultural and developmental aspects, highlighting the complex psychiatric outcomes traumatized adolescents may face. Researchers in the study [29] touch upon the necessity of adapting interventions to the developmental level of the child or adolescent for effective treatment. Furthermore, researchers in the study [49,52] showcase how cultural contexts can influence treatment outcomes, demonstrating the effectiveness of trauma-focused interventions in diverse settings like South African schools. Researchers in the study [42] stress the role of cultural sensitivity in psychotherapeutic treatments, particularly for refugee children, suggesting that incorporating non-verbal communication elements like art-based interventions can be beneficial. The researcher in the study [39] discusses the need to tailor TF-CBT to the specific developmental and cultural needs of patients for its efficacy. The research team in the study [48] highlight the necessity of culturally sensitive interventions for children from war-torn regions. Together, these discussions underscore the crucial role of cultural and developmental considerations in tailoring psychotherapeutic interventions to maximize efficacy in treating PTSD in children and adolescents, influencing not only therapy choices, but also their implementation and expected outcomes.

### 5.4. Research Question 4 [RQ4]

[RQ4] investigates the long-term effects and sustainability of psychotherapeutic interventions on PTSD symptoms in children and adolescents, with several studies contributing valuable insights. Researchers in the study [41], demonstrate the enduring benefits of TF-CBT, showing sustained symptom relief and improved psychosocial functioning over extended periods. Researchers in the study [42] find that narrative exposure therapy (NET) provides long-lasting benefits, reducing PTSD symptoms and psychological distress while enhancing resilience in multiply traumatized youth. Also, researchers in the studies [49,52] highlight the sustained improvements in PTSD and depression symptoms with Prolonged Exposure Therapy for Adolescents (PE-A) up to 12 months post-treatment. Researchers in the study [30] discuss the sustained benefits of a targeted preventive intervention in reducing PTSD symptom severity over time, particularly emphasizing early intervention for young children. Furthermore, researchers in the study [29] affirm the sustainable long-term benefits of TF-CBT in significantly reducing PTSD symptoms compared to control treatments. Two studies [31,39] emphasize the importance of sustaining improvements in PTSD symptoms over time through interventions like TF-CBT and Cognitive Behavioral Intervention for Trauma in Schools (CBITS). Also, researchers in the study [36] provide insights into the sustainable benefits and cost-effectiveness of Cognitive Therapy for PTSD (CT-PTSD) in children and adolescents. Together, these studies underscore the enduring effects of various psychotherapeutic interventions in reducing PTSD symptoms and enhancing overall psychological resilience and functioning in affected youth, highlighting the importance of selecting therapies that contribute to long-term well-being.

### 5.5. Research Question 5 [RQ5]

[RQ5] aiming to determine the most effective psychotherapeutic interventions for different age groups within the pediatric PTSD population is addressed by several studies. Researchers in the study [34] assess TF-CBT’s effectiveness across various adolescent age groups in real-world urban community settings, offering insights into its practical application. Furthermore, researchers in the study [21] potentially cover the effectiveness of psychological treatments for children and adolescents with PTSD, including an analysis by age group, focusing on therapies like CBT and EMDR. Researchers in the study [45] delve into TF-CBT within specific adolescent age ranges, evaluating its effectiveness in reducing PTSD symptoms tailored to particular age groups. Moreover, researchers in the study [38] explore life skills programs for adolescents, shedding light on their effectiveness for different age groups within the pediatric population and demonstrating how psychotherapeutic interventions can be adapted based on developmental stages. Although researchers with their study [28] may not explicitly address different age groups, their study on trauma’s differential impacts on children and adolescents following an earthquake could inform tailored psychotherapeutic interventions for different ages. Researchers in the study [46] investigate the impact of an integrative therapy program, including mindfulness and arts, for adolescent girls, potentially providing insights into age-appropriate interventions effective for older children in the pediatric group. These studies collectively offer valuable insights into the effectiveness of various psychotherapeutic interventions tailored to specific age groups within the pediatric PTSD population, encompassing cognitive-behavioral strategies to more integrative techniques like mindfulness and arts.

Also, ref. [RQ5] pertains to the comparative long-term effects of EMDR versus CBT in treating pediatric PTSD, along with factors influencing their sustainability. Several studies from the provided list contribute to addressing this question. Specifically, researchers in the study [21], while not explicitly focused on EMDR versus CBT, may offer insights into the long-term effects of different psychotherapeutic interventions for pediatric PTSD and factors influencing sustainability. Similarly, researchers in the study [45] investigated TF-CBT, closely related to CBT, potentially shedding light on the long-term effects and sustainability of CBT-based interventions. Additionally, in the study [34], researchers, benchmarking TF-CBT’s effectiveness in urban community settings, might provide insights into the long-term effects of CBT-based interventions for pediatric PTSD and factors influencing sustainability. The research project [35] explored the long-term effects of Internet-based CBT for children with anxiety disorders, offering insights into CBT-based interventions’ long-term effects and sustainability in pediatric populations. Additionally, in the study [38], researchers investigated life skills programs targeting mental health outcomes in adolescents, potentially offering insights into the long-term effects and sustainability of psychotherapeutic interventions tailored to pediatric populations. While these studies may not directly compare EMDR and CBT, they provide valuable insights into the long-term effects of psychotherapeutic interventions and factors influencing their sustainability in treating pediatric PTSD.

### 5.6. Research Question 6 [RQ6]

[RQ6] investigates how developmental stages affect the choice and success of psychotherapeutic interventions for PTSD in children and adolescents. Several studies, found in the list in Table 1 of the present study, contribute to addressing this question. Specifically, the researchers in the study [21] may offer insights into how various psychotherapeutic interventions are adapted to suit the developmental needs of children and adolescents with PTSD, although not explicitly focused on developmental stages. Researchers in the study [45], focusing on TF-CBT in reducing PTSD symptoms in adolescents, could shed light on interventions tailored to the developmental stage of adolescents and their effectiveness. Additionally, researchers in the study [35], although examining Internet-based CBT for children with anxiety disorders, might offer insights into adapting interventions for different developmental stages and their success in treating pediatric mental health conditions. Similarly, in the study [38], researchers exploring life skills programs targeting mental health outcomes in adolescents could provide insights into interventions tailored to adolescents’ developmental needs and their effectiveness in improving mental health outcomes. Additionally, researchers in the study [43], investigating the effectiveness of narrative exposure therapy for children and adolescents with PTSD, might offer insights into interventions’ adaptation for different age groups and their success in treating PTSD symptoms. While these studies may not directly address the influence of developmental stages on psychotherapeutic interventions for PTSD, they provide valuable insights into how interventions are tailored to different age groups and their effectiveness in treating PTSD symptoms.

The study of PTSD in children and adolescents has advanced significantly in recent years. Several interventions can aid in symptom management. These may be psychological or pharmacological. It is essential to mention that the specific requirements of clinical or psychotherapeutic interventions addressing child and adolescent disorders linked to traumatic experiences are different from those applied to adults. However, there is a gradual increase in the quality and quantity of research on young individuals. It is now evident that children and adults behave and think differently. This distinction will aid in identifying and comprehending the unique requirements of each age group, resulting in more effective treatment and prevention of their problems. Without doubt, child and adolescent PTSD and psychopathology research has a long way to go.

As demonstrated by the preceding comparison, CBT, specifically trauma-focused CBT, is the treatment of choice for pediatric and adolescent PTSD. Multiple researchers have studied it, and it is now substantiated by evidence. CBT is a broad category encompassing various methods and approaches, such as exposure therapy and relaxation techniques. Numerous researchers support EMDR therapy, which is quite convincing. Systems therapy, play therapy, psychodynamic psychotherapy, etc., are additional varieties of therapy. In addition to psychological remedies, there are also pharmacological treatments, which are widely available but should be used with caution.

One crucial challenge for the effectiveness of clinical psychological and psychotherapeutic interventions is to systematically investigate the long-term effect of the treatment, as well as to evaluate the global psychosocial thrive and development of subjects who have suffered from a severe trauma, alongside symptom reduction. It also imperative to explore the “*what and why it works*” in each intervention and analyze, for each psychotherapeutic treatment, the specific factors that contribute to the therapeutic effect [53,54]. It could be, for example, assumed that the efficacy of the CBT model does not exclusively lie in the possibility of modifying the negative intrusive thoughts, but also in the fact of providing, to a traumatized subject, a very structured model of intervention alongside the active involvement of the therapist/clinical in the treatment process. 

It should not, however, be overlooked the fact that the efficacy of the treatment can be affected by multiple variables, including the duration and nature of the trauma, the family environment and parental response to treatment, the age of the subject, the previous psychosocial development, and personality traits (e.g., quality of defenses/coping mechanisms, etc.), as well as factors related to specific conditions of applying treatment. Typically, this factor is not considered when conducting research. The involvement of parents and caregivers is an important facilitating factor that may impact the effectiveness of treatment. According to [23,24], a meta-analysis, parental involvement improves treatment efficacy. Individual therapies appear more efficient than group therapies.

Furthermore, the COVID-19 pandemic has exerted a substantial influence on the mental well-being of children and adolescents, with specific subgroups being especially susceptible to the psychological consequences of the pandemic [55]. This highlights the necessity for comprehensive interventions to tackle PTSD and associated mental health problems amid worldwide emergencies. Furthermore, there is a proposal to develop and pilot test a traumatic stress screening tool specifically designed for adolescents in pediatric primary care [56]. The goal of this initiative is to enhance the detection and treatment of traumatic stress in this particular group [57].

Understanding the nuanced and complex nature of Post-Traumatic Stress Disorder (PTSD) in children and adolescents necessitates a specialized approach to clinical interventions that takes into consideration not only the symptoms, but also the unique developmental, psychological, and social/family contexts of this population. This paper has synthesized a broad spectrum of contemporary research findings, spanning from 2016 to 2023, to highlight the prevalence of PTSD among young populations, delineate the distinguishing characteristics of PTSD symptoms across different age groups, and critically assessed the efficacy of various therapeutic interventions.

Research unequivocally points to PTSD as a significant concern among children and adolescents who have been exposed to traumatic events. The prevalence of PTSD within these age groups suggests a distressing psychosocial phenomenon, with symptoms manifesting divergently based on developmental stages. For example, younger children, aged 6–12, might experience a disturbed sense of time, engage in post-traumatic play that re-enacts the trauma, and suffer from sleep disturbances, such as nightmares. Adolescents, on the other hand, tend to exhibit symptoms like dissociative behaviors, anger outbursts, self-injurious actions, and substance abuse, reflecting a more complex cognitive and emotional processing of trauma.

Diagnostic assessments of PTSD in younger populations also face unique challenges, including difficulties in defining what constitutes a traumatic event for this demographic, cognitive limitations that impede the expressive capability regarding their experiences, and a general reluctance to discuss traumatic events due to fear or avoidance tendencies [58]. These diagnostic complexities necessitate therapeutic interventions that are not only robust and evidence-based, but are also flexible and sensitive to the individual needs and developmental stages of children and adolescents.

Among the array of therapeutic interventions analyzed, CBT and specifically TF-CBT emerge as the most effective and extensively employed methodologies for addressing PTSD in children and adolescents. These approaches combine cognitive and behavioral strategies to mitigate PTSD symptoms regardless of trauma type, with TF-CBT incorporating additional elements like cognitive processing, family involvement, and exposure therapy. Conversely, EMDR and Narrative Exposure Therapy (NET) offer alternative, yet comparably effective, treatments that focus on desensitizing patients to traumatic experiences and reconstructing personal narratives of trauma, respectively.

As for the psychodynamic treatment, it is worth noticing that in recent years, more studies have focused on children who experience severe parental conflict and/or domestic violence [35]. The study [59] suggests that children experiencing trauma symptoms are mainly able to benefit from group psychodynamic therapy, suggesting a promising area for future research with children impacted by parental conflict. 

In addition, eight studies, including three RCTs, have evaluated the effectiveness of psychodynamic therapies with children who had experienced trauma, including children in foster care and post-adoption. These findings are promising and show that psychodynamic therapy is as effective as alternative treatments [60]. However, the conclusion of the systematic review [40] is that there is an urgent need to build on the preliminary research in this area with more extensive and better-designed studies.

CBT has garnered significant recognition as an efficacious intervention for PTSD among a diverse range of individuals. For the treatment of PTSD in children and adolescents, trauma-focused CBT is effective [61]. Furthermore, there is substantial evidence supporting the efficacy of TF-CBT delivered by therapists in the treatment of PTSD [62]. Additionally, Internet-delivered CBT and i-CBT are effective interventions for adults with PTSD, according to studies [63]. Moreover, sleep disturbances among veterans with PTSD have been effectively reduced through CBT [64].

CBT for PTSD is more effective than treatment as usual or unstructured therapy modalities, according to empirical data [65], producing clinically significant outcomes. Furthermore, empirical research indicates that CBT is efficacious when customized to address specific concerns, solidifying its reputation as the treatment of choice for PTSD [66]. Consistent with the literature, CBT is a safe and productive intervention for acute and chronic PTSD in children, adolescents, and adults [67].

Studies have shown that CBT is effective in the post-treatment phase of PTSD, indicating that its effects endure [68]. In addition, cognitive control network activity is increased in patients with major depression and PTSD, suggesting that CBT may have a beneficial effect on the severity of symptoms [69]. Additionally, research has demonstrated that cognitive-behavioral therapy (CBT) is viable and correlated with ameliorations in symptoms and associated results among people who also suffer from severe mental illness and borderline personality disorder [70].

Additionally, multiple studies have established EMDR as an efficacious therapeutic approach for PTSD. EMDR therapy employs eye movements to elicit orientation responses (ORs), which assist individuals in forming adaptive associations between adverse experiences and positive emotions and cognitions. This process ultimately results in a substantial amelioration of symptoms associated with PTSD [71]. Scholarly investigations have demonstrated that EMDR therapy exhibits a markedly superior efficacy in ameliorating symptoms of PTSD in comparison to control conditions and alternative interventions, such as CBT [72].

Research has shown that EMDR is effective in alleviating symptoms of PTSD across a range of populations, including adults and Syrian refugees [73,74]. In addition, EMDR reduces the severity of PTSD symptoms more effectively than brief eclectic psychotherapy [75], with a more pronounced decline in symptoms. In addition, when compared to waitlist conditions, EMDR has demonstrated efficacy in mitigating symptoms of PTSD in children, yielding results comparable to those of CBT [76].

Furthermore, the efficacy of EMDR therapy in mitigating symptoms of PTSD among individuals with intellectual disabilities has been emphasized, implying that it might surpass the effectiveness of verbal interventions tailored to this population [77,78]. Furthermore, promising results in EMDR’s ability to alleviate PTSD symptoms have been observed in a variety of settings, including the treatment of PTSD in pregnant women and postpartum [79,80]. Further research indicates that EMDR therapy has the potential to treat a variety of psychological conditions, including personality disorders that do not involve PTSD [81,82].

Additionally, alternative therapies like art-based interventions and other culturally sensitive approaches should be considered to address the unique requirements of individuals from diverse backgrounds. However, additional research is required to investigate the influence of therapist traits, family factors, and treatment adherence on treatment outcomes. 

The comprehensive systematic review of clinical interventions for PTSD in children and adolescents reveals several critical insights regarding the quality of the studies included and the potential heterogeneity among them. The review primarily focuses on interventions like CBT, TF-CBT, EMDR, and various others. The quality of these studies is generally essential, with many employing rigorous methodologies such as randomized controlled trials (RCTs). This approach strengthens the reliability of the findings, suggesting that therapies like TF-CBT and EMDR are effective for treating PTSD in children and adolescents. Furthermore, including meta-analyses and systematic reviews further enhances the quality of the evaluation by aggregating data from multiple studies, allowing for more robust conclusions about the efficacy of various interventions. It is important to note that the studies included in the review typically used established diagnostic criteria (DSM-5 or ICD-11) and standardized outcome measures for PTSD, which adds to the reliability of the findings. However, the variability in outcome measures across studies could contribute to heterogeneity. Also, the review covers a wide range of psychotherapeutic interventions (e.g., TF-CBT, EMDR, play therapy), which inherently differ in approach and implementation. This diversity can lead to heterogeneity in outcomes, as different treatments may be variably effective depending on the specific needs and backgrounds of the children and adolescents treated. The age range of participants (5 to 17 years old) encompasses a broad developmental spectrum from early childhood to late adolescence. This broad age range can introduce heterogeneity due to developmental differences in understanding, processing, and coping with trauma. The review likely includes studies with participants who have experienced different types and severities of trauma (e.g., abuse, natural disasters, war). The heterogeneity in trauma experiences can affect treatment outcomes, as some interventions may be more suitable for certain types of trauma than others. Additionally, the studies included in the review may vary in their participants’ cultural and socioeconomic backgrounds. These factors can influence the presentation of PTSD symptoms and the effectiveness of interventions, contributing to heterogeneity in outcomes. There may be variability in the duration of treatments and the mode of delivery (individual vs. group therapy, in-person vs. online) across the studies. These differences can lead to variations in treatment outcomes. Children and adolescents with PTSD often have comorbid conditions (e.g., anxiety, depression, behavioral disorders). The presence and treatment of comorbidities can introduce additional variability in outcomes. The quality of the studies included in the review is generally high, providing valuable evidence for the efficacy of various interventions for PTSD in children and adolescents. However, potential heterogeneity among the studies can arise from differences in interventions, participant characteristics, trauma types, cultural backgrounds, treatment modalities, and comorbid conditions. Understanding this heterogeneity is crucial for tailoring interventions to meet the individual needs of children and adolescents affected by PTSD.

Amplifying the complexity of treating PTSD in youth are factors such as gender differences, with emerging evidence suggesting a heightened risk of PTSD diagnosis in females and the crucial role of parental involvement in the therapeutic process. Treatment duration, therapist characteristics, and the necessity for tailored interventions based on the child’s cultural background further underscore the multifarious considerations required for effective treatment.

As separate research on PTSD in children and adolescents began relatively recently, the current literature on youth PTSD is insufficient. It would be beneficial to investigate various types of trauma separately to determine whether specific treatments are more effective for certain types of trauma. Most existing meta-analyses combine all forms of trauma, obscuring potential differences in treatment outcomes. In addition to the form of trauma, it would be beneficial to differentiate between PTSD resulting from a traumatic event and PTSD resulting from chronic, repeated traumatic factors. The mechanisms implicated in the two cases may differ, necessitating a different approach. Additionally, it would be beneficial to contemplate more severe co-occurring disorders. According to the research, PTSD frequently coexists with other disorders (anxiety, melancholy, etc.), which may hinder the recovery of children. A more extensive survey sample could aid in consolidating and generalizing the findings.

The present systematic review has some limitations that should be mentioned. First, the review has not adequately addressed how cultural and geographical factors affect the efficacy of different therapies, which is particularly important given the variability in PTSD presentations across different cultural contexts. Second, there was no mention of assessing for publication bias, which could influence the findings of studies with positive outcomes more likely to be published than those with negative or inconclusive results. Third, there was variability in intervention effectiveness. For instance, the excluded studies have explored different interventions, combinations of therapies, or have involved diverse demographic groups. Discussing these limitations can help contextualize the findings, acknowledge the scope of the review, and guide future research to address these gaps effectively.

As previously stated, childhood and adolescence psychopathology research is limited and has a long way to go. In addition to the limited quantity of empirical research, we frequently encounter research of “low” quality. Most research is not grounded in empirical evidence or randomized controlled trials. In addition, the majority of studies employ tiny samples. Spontaneous remission, or “resolution”—improvement of the disorder’s clinical symptoms without external treatment—has not been measured in most studies. This would be beneficial in determining what proportion of the improvement is attributable to the intervention used each time and what proportion is attributable to spontaneous remission over time. Without doubt, there are significant gaps in childhood and adolescence psychopathology research, particularly noting the scarcity and often low quality of empirical studies. This shortfall is especially concerning when considering complex psychological phenomena such as cognitive dynamics, trauma, psychosis, and personality disorders [83]. These elements are profoundly interlinked, influencing and exacerbating each other in subtle and often unpredictable ways. For instance, trauma experienced in early life can alter cognitive dynamics, potentially leading to the development of psychosis and personality disorders as coping or defense mechanisms [84]. The lack of robust, large-scale, and controlled studies makes it challenging to discern the natural course of these disorders and the specific impacts of interventions. Furthermore, the failure to measure spontaneous remission leaves a significant knowledge gap in our understanding of how these disorders might resolve independently of interventions. This makes it difficult to evaluate the true efficacy of treatments, thereby complicating clinical decisions and policy-making aimed at effectively addressing these complex disorders in young populations.

To sum up, this paper underscores the imperative for an individualized, culturally sensitive approach to treating PTSD in children and adolescents. It advocates for a holistic understanding that integrates the symptomatic, developmental, and social dimensions of the disorder, thereby paving the way for interventions that are not only effective, but also empathetic to the unique challenges faced by young individuals recovering from trauma. The ongoing research and refinement of therapeutic strategies remain crucial in ensuring that all affected children and adolescents have access to evidence-based, developmentally appropriate care.

**Table 1:** Main Results and Study Characteristics.

Author (Year)	Type of Study	Sample (N)	Assessment/Intervention	Main Findings/Outcomes
Ben Ari et al. (2019) [26] [RQ1]	Prospective Study	Children N = 151	Semi-structured interview CBCL (child behavior checklist) PTSDSSI (post-traumatic stress disorder semi-structured interview) PCASS (the Preschool Children’s Assessment of Stress Scale) UCLA-PTSD (University of California at Los Angeles PTSD) reaction index: DSM-5 version SCARED (the screen for child anxiety related emotional disorders)	Medical information exposure inversely affects post-traumatic stress in children months after a medical episode. The correlation is significant in preschoolers and schoolchildren.
Budde et al. (2018) [27] [RQ5]	Quantitative	Adolescents aged 13–16 years N = 198	Impact of an 8-week exercise training (ET) regime on PTSD symptoms and changes in cortisol levels.	Exercise training may affect cortisol levels, introduce a cost-effective group intervention for PTSD patients, and differ from a placebo.
Chen et al. (2018) [85] [RQ5]	Quantitative	Adolescents aged 12–16 years N = 251	EMDR	- EMDR reduced PTSD, depression, and anxiety more than other therapies and controls. - Clinical efficacy of EMDR in treating complex childhood trauma in children and adults.
Danielson et al. (2020) [47] [RQ1] [RQ2]	Quantitative	Adolescents aged 13–17 years [RRFT: 61] [TAU: 63] N = 124	The intervention(s) that the study participants received were Risk Reduction through Family Therapy (RRFT) and Treatment-As-Usual (TAU) consisting primarily of trauma-focused cognitive behavioral therapy. RRFT resulted in significantly greater reductions in substance-using days at month 12 and month 18 compared to TAU. Significant reductions in PTSD symptoms were observed within the RRFT group at months 3, 6, 12, and 18.	- RRFT reduced substance-using days more than the control group at months 12 and 18. - RRFT and TAU groups showed significant PTSD symptom reductions from baseline to months 3, 6, 12, and 18, with no between-group differences. - Neither condition showed worsening substance use problems.
Danzi & La Greca (2017) [21] [RQ5] [RQ6]	Longitudinal	37 studies on PTSD, 20 RCTs on PTSD, 41 RCTs of varied interventions for youth with PTSD, 135 studies on psychological treatments for PTSS in youth and found the largest effect sizes for CBT	Semi-structured interview and questionnaires examining these parameters: Gender, Age, Ethnicity, Domicile, Parent/Caregiver Factors, Trauma Types, Treatment Factors.	Six recent meta-analyses and systematic reviews examined PTSD psychological treatments for children and adolescents. CBT, EMDR, narrative exposure therapy, and classroom-based interventions were supported. CBT and TF-CBT are well-established treatments for youth PTSD. Despite limited evidence, EMDR, narrative exposure therapy, and school-based interventions gained support.
El-Khodary et al. (2019) [48] [RQ1] [RQ3]	Quantitative	Children and Adolescents 11–17 years old N = 1029	War-Traumatic Events Checklist (W-TECh), Multicultural Events Schedule for Adolescents (M.E.S.A.) Post-traumatic Stress Disorders Symptoms Scale (PTSDSS) Strengths and Difficulties Questionnaire Child Depression Inventory (CDI)	At least one war-traumatic event affected every child or adolescent, worsening mental health and behavior. These families and children need counseling.
Ferrajão (2020) [50] [RQ2]	Quantitative	Children N = 60	Child PTSD Symptom Scale Children’s Depression Inventory 2 Emotional Validation Experiences Questionnaire	This population may benefit from clinical intervention goals like parental emotional validation and invalidation. Therapy interventions suggest emotional validation to improve therapist-patient relationships.
Forresi et al. (2019) [28] [RQ5]	Cross-sectional	Children and Adolescents 9–14 years N = 682 Parents N = 1162	UCLA PTSD-Index Strengths and Difficulties Questionnaire (SDQ) SCL-90	The findings suggest better clinical interventions for earthquake-exposed children and adolescents.
Grainger et al. (2022) [29] [RQ1] [RQ3] [RQ4]	Systematic Review & Meta-analysis	40 RCTs	PROSPERO TF-CBT interventions	The results showed that TF-CBT reduced PTSD symptoms better than controls.
Haag et al. (2020) [30] [RQ4]	Quantitative	Participant age: 1–6 years [Intervention: 62] [Treatment-as-usual: 71] N = 133	The intervention was a 2-session CBT-based intervention for parents of children who screened ‘high-risk’ for PTSD. The duration of the intervention was 2 sessions.	The targeted preventive intervention reduced posttraumatic stress disorder (PTSD) symptom severity over time, with intervention children having a faster PTSS severity score reduction than controls at 3 months. The intervention also improved PTSD diagnosis, functional impairment, and behavioral issues in young injured children.
Kolaitis, G. (2017) [31] [RQ4]	Systematic Review	Children and Adolescents	The interventions that the study participants received include psychoeducation about trauma reactions, exposure to trauma-related cues and memories, coping skills training for children, parental training, and medications such as selective serotonin reuptake inhibitors.	The paper reviews predictors, assessment, and treatment options for youth with PTSD, suggests studying phenotypes or domains like cognitive, memory, and executive functioning to understand PTSD and its effects, and proposes a dimensional approach to PTSD and trauma.
Luoni et al. (2018) [86] [RQ3]	Cross-sectional	Adolescents between 12 and 18 years N = 107	Wechsler Intelligence Scale for Children-IV Minnesota Multiphasic Personality Inventory–Adolescent Version Trauma Symptom Checklist for Children (form TSCCA) Child Behavior Checklist (Achenbach) Clinical Global Impressions-Severity of Illness Scale	Traumatized adolescents can develop short- and long-term psychiatric diagnoses like affective, personality, and psychotic disorders, as well as dissociative and somatic symptoms that may be more debilitating than PTSD. Trauma treatment requires individualization.
Márquez et al. (2020) [32] [RQ1]	Qualitative	Case Study 14-year-old Guatemalan female N = 1	Trauma-Focused Cognitive Behavioral Therapy (TF-CBT) PRACTICE—Psychoeducation & Parenting skills, Relaxation, Affective expression and modulation, Cognitive coping, Trauma narrative and processing, In vivo mastery, Conjoint sessions, and Enhancing safety and future development	Youth at risk for familial sex trafficking and labor exploitation may benefit from TF-CBT for psychosocial issues. TF-CBT can help traumatized children cope and improve their mental health.
Mavranezouli et al. (2019) [33] [RQ1]	Systematic Review	Adolescent aged < 18 years	Cognitive therapy for PTSD, narrative exposure, play therapy, other forms of individual TF-CBT, EMDR, parent training, group TF-CBT, family therapy, supportive counselling.	- Cognitive therapy for PTSD in children and youth is the most cost-effective, followed by narrative exposure, play therapy, and other individual TF-CBT. - Individual TF-CBT and play therapy are cost-effective for children and youth with PTSD more than 3 months after trauma. - Family therapy and supportive counseling may not be cost-effective over other interventions.
Miodus et al. (2021) [44] [RQ1]	Quantitative	College Students < 18 N = 454	UCLA PTSD Reaction Index, DSM-IV The Barkley Adult ADHD Rating Scale–IV Beck Depression Inventory–Second Edition Beck Anxiety Inventory	Childhood ADHD symptoms were linked to trauma exposure and PTSD in college students. Clinical interventions for children and adolescents, college counseling, and psychological and academic inclusion services are impacts.
Nöthling et al. (2016) [87] [RQ1]	Quantitative	Adolescents N = 215	Kiddie Schedule for Affective Disorders and Schizophrenia (K-SADS-PL) Child PTSD Checklist (CPC) Childhood Trauma Questionnaire (CTQ) Child Exposure to Community Violence Checklist (CECV)	Interventions should address individual/interpersonal (reducing home and environmental abuse) and community/societal (reducing crime rates and strengthening conviction policies) levels to prevent trauma, PTSD, and depression.
Peltonen, K., & Kangaslampi, S. (2019) [42] [RQ1] [RQ3] [RQ4]	Quantitative	Children and Adolescents 9–17 years [NET: 29] [TAU: 21] N = 50	NET Questionnaire	The study found that narrative exposure therapy (NET) and treatment as usual (TAU) reduced PTSD, psychological distress, and rebuilt resilience. PTSD symptoms and clinical-level PTSD rates decreased significantly in the NET group only, with large effect sizes. The study suggests that NET is safe, effective, and useful for multiply traumatized children and adolescents in clinical settings.
Peters et al. (2021) [45] [RQ5] [RQ6]	Quantitative	Adolescents aged < 18 N = 20	The intervention received by the study participants is TF-CBT, with a mean of 15 sessions over 25 weeks.	The study found that TF-CBT significantly reduced PTSD symptoms, with only 1 out of 16 participants meeting diagnostic criteria after treatment. The self-report measures of PTSD, anxiety, and depression improved significantly. Some participants experienced a brief exacerbation in symptoms during certain phases of the treatment, but all symptoms resolved by the end, and the majority of participants rated the intervention as helpful.
Roque-Lopez et al. (2021) [46] [RQ5]	Quantitative	Girls aged 13–16 years N = 44	Adverse childhood experience (ACE) Short PTSD Rating Interview (SPRINT) Child PTSD Symptom Scale (CPSS) Mindful Attention Awareness Scale—Adolescents (MAAS-A)	The intervention included mindfulness, expressive arts, and EMDR group therapy. This integrative/complementary short-term program may reduce psychological burden in adolescents with multiple adverse childhood experiences. After 2 months, adolescents’ psychological functioning improved, but they may need group or individual follow-up to maximize its mental health benefits.
Rossouw et al. (2016) [52] [RQ1] [RQ3] [RQ4]	Quantitative	Adolescents aged 14–18 years [PE-A: 6] [SC: 5] N = 11	The intervention(s) that the study participants received were: 1. Prolonged Exposure Treatment for Adolescents (PE-A): up to 14 weekly 60 to 90 min sessions, comprising eight modules with specific activities and homework exercises. 2. Supportive Counselling (SC): up to 14 weekly 60 to 90 min sessions, focusing on client-centered therapy and establishing a trusting therapeutic relationship.	Both PE-A and SC improved PTSD and depression symptoms during treatment, according to the study. PE-A may be long-lasting because participants in the group maintained their PTSD and depression gains at the 12-month post-treatment assessment. The study also showed that inexperienced school counselors in South Africa can implement the PE-A protocol.
Rossouw et al. (2018) [49] [RQ1] [RQ3] [RQ4]	Quantitative	Adolescents aged 13–18 [PE-A: 31] [Supportive Counselling: 32] N = 63	Prolonged exposure therapy for PTSD: 7–14 weekly, 60 min sessions with eight modules and homework exercises. Supportive counselling: 7–14 weekly, 60 min sessions of client-centered therapy.	At post-intervention, 3-month, and 6-month follow-up assessments, prolonged exposure (PE-A) reduced PTSD symptoms in adolescents more than supportive counselling. - More PE-A participants had a ‘good response’ than supportive counselling participants, indicating a higher rate of positive treatment outcomes. PE-A reduced PTSD symptoms and increased remission rates in adolescent PTSD more than supportive counselling.
Rudd et al. (2019) [34] [RQ5]	Quantitative	Children N = 114	Child PTSD Symptom Scale Ohio Mental Health Consumer Outcomes System—Ohio Youth Problem, Functioning, and Satisfaction Scales	This study is the first benchmarking study of TF-CBT and provides preliminary findings on its efficacy and transportability to urban community settings serving poor youth.
Russotti et al. (2023) [51] [RQ2]	Longitudinal Cohort	Adolescent Females 15–17 years N = 514	Child maltreatment determined by substantiated caseworker reports Beck Depression Inventory-II Comprehensive Trauma Interview Inventory of Parent and Peer Attachment—IPPA Child’s Report of Parental Behavior Inventory	The current study used a person-centered approach to (a) identify subgroups of adolescent females with distinct attachment quality with peers, fathers, and mothers and (b) determine if maltreatment affected depressive and PTSD symptoms differently by attachment quality.
Sarkadi et al. (2017) [35] [RQ5] [RQ6]	Qualitative	Adolescent N = 139	Teaching Recovery Techniques (TRT)—6 week program	Despite 62% of participants experiencing negative life events during the program and being in asylum, pre- and post-measures showed significant differences in depressive and PTSD symptoms. Six qualitative interview categories were social support, normalization, valuable tools, comprehensibility, manageability, and meaningfulness. According to TRT’s program theory, sharing experiences in a safe and supportive environment and learning trauma-specific exposure and behavioral activation will help youth feel more coherent and reduce depression and PTSD symptoms. URMs may benefit from TRT for PTSD.
Shearer et al. (2017) [36] [RQ4]	Quantitative	Children and Adolescents aged 8–17 years N = 29	Cognitive therapy for PTSD (CT-PTSD)—Individual weekly sessions over 10 weeks delivered by trained clinical psychologists. Average contact time: 636.25 min. Average number of sessions: 8.3. Mean cost: £1463.	Cognitive therapy is likely cost-effective compared to usual care for children and adolescents with PTSD, according to the study.
Shi et al. (2018) [37] [RQ2]	Quantitative	Adolescents: 15.22 years Parents: 41.04 years N = 688	PTSS Questionnaire	The study highlights the mutual impacts of adolescent and parental PTSS after a disaster, with both maternal and paternal PTSS at 12 months predicting adolescent PTSS at 18 months. Adolescent PTSS at 12 months only predicted maternal PTSS at 18 months, not paternal PTSS.
Singla et al. (2020) [38] [RQ5] [RQ6]	Quantitative	Adolescents aged 10–19 years	The study participants received life skills programs targeting one or more mental health outcomes and co-occurring risk factors in school and community settings. The interventions were delivered by teachers and specialist providers, focused on high-risk groups, and included comprehensive programs focusing on multiple life skills related to the individual, their social environment, and interventions promoting parent–child interactions.	Most RCTs on LMIC students and refugees reduced anger, improved life skills and functioning, and reduced PTSD, depression, and anxiety. LMICs may benefit from comprehensive programs that address multiple life skills, the individual’s social environment, and parent–child interactions to address mental health and other health issues.
Syros, I. (2017) [39] [RQ1] [RQ3] [RQ4]	Quantitative	Child and Adolescent aged between 3–17 years N = 900	TF-CBT: - Demonstrated clinically significant improvement in more than 900 children. - Follow-up studies show sustainability of benefits for 6 months, 1 year, and 2 years after treatment. CBITS: - Superior to the waiting list in reducing PTSD and depression cases.	Most effective PTSD treatments include CB, and TF-CBT and CBITS have shown clinically significant improvement and sustained benefits.
Turrini et al. (2019) [40] [RQ1]	Quantitative	Children from 7 years N = 1959	NET, KIDNET, EMDR, music therapy, CETA, CBT, writing for recovery, IPT, TRT, CROP, FGI, need satisfaction intervention.	Clinically significant psychosocial interventions improve PTSD, depression, and anxiety in distressed refugees and asylum seekers. Most evidence supports trauma-focused cognitive behavioral therapies. Accordingly, develop evidence-based guidelines and implementation packages.
Tutus et al. (2017) [41] [RQ4]	Quantitative	Mean age: 12.66 years N = 76	Intervention: - Trauma-focused cognitive-behavioral therapy (TF-CBT). - Additional interventions during the follow-up period: psychotherapy, counseling, rehabilitation, consultation with a psychiatrist, and psychotropic medication.	TF-CBT is effective in reducing posttraumatic stress symptoms and improving psychosocial functioning over the long term.
van der Spuy et al. (2018) [43] [RQ6]	Quantitative	Children aged 5–7 years N = 12	Trauma Symptom Checklist for Young Children (TSCYC)	Eye Movement Integration (EMI) reduced all but one post-traumatic stress symptom in young children in resource-constrained settings.

## 6. Conclusions

In conclusion, PTSD in children and adolescents is a complex mental health issue requiring comprehensive clinical interventions. Post-traumatic Stress Disorder (PTSD) in children and adolescents is a severe, potentially debilitating condition that can interfere with their development and well-being as a whole. It is triggered by exposure to traumatic events, which can be experienced directly or witnessed. Early identification and intervention are essential for preventing the long-term mental, emotional, and even physical health effects that this condition can have on children. The effectiveness of psychotherapeutic interventions in treating PTSD in this population has been demonstrated. CBT, TF-CBT, and EMDR are among the therapies with the most robust empirical support. They assist children and adolescents in processing traumatic events, reducing distressing symptoms, and enhancing coping mechanisms. Specifically, TF-CBT has been extensively studied and has demonstrated remarkable efficacy in reducing PTSD symptoms and improving children’s and adolescents’ functioning and quality of life. It integrates trauma-sensitive interventions with cognitive behavioral therapy techniques to assist adolescents in comprehending and managing their emotional reactions to traumatic events. Additionally, involving the parents or caregivers in the therapeutic process is essential. Family support can substantially improve treatment efficacy and facilitate the child’s recovery. However, treatment must be tailored to the child’s age, developmental level, specific symptoms, and the nature of the trauma. Not all children and adolescents respond the same way to treatment; therefore, it is necessary to monitor and modify treatment plans. PTSD in children and adolescents is a significant public health concern requiring trauma-informed, comprehensive care. Psychotherapeutic interventions, in particular TF-CBT, are highly effective for symptom management and reduction. However, additional research is required to ensure that all affected children and adolescents have access to evidence-based treatment. Public awareness and education about PTSD in adolescents and its treatment options are essential for early detection and intervention.

## Data Availability

Not applicable.
